# A Soft Reconfigurable Inverted Climbing Robot Based on Magneto-Elastica-Reinforced Elastomer

**DOI:** 10.3390/mi16080855

**Published:** 2025-07-25

**Authors:** Fuwen Hu, Bingyu Zhao, Wenyu Jiang

**Affiliations:** 1 School of Mechanical and Material Engineering, North China University of Technology, Beijing 100144, China; 2 Brunel London School, North China University of Technology, Beijing 100144, China

**Keywords:** magneto-elastica, soft robot, reconfigurable robot, inverted climbing, omnidirectional motion

## Abstract

This work presents a novel type of soft reconfigurable mobile robot with multimodal locomotion, which is created using a controllable magneto-elastica-reinforced composite elastomer. The rope motor-driven method is employed to modulate magnetics–mechanics coupling effects and enable the magneto-elastica-reinforced elastomer actuator to produce controllable deformations. Furthermore, the 3D-printed magneto-elastica-reinforced elastomer actuators are assembled into several typical robotic patterns: linear configuration, parallel configuration, and triangular configuration. As a proof of concept, a few of the basic locomotive modes are demonstrated including squirming-type crawling at a speed of 1.11 mm/s, crawling with turning functions at a speed of 1.11 mm/s, and omnidirectional crawling at a speed of 1.25 mm/s. Notably, the embedded magnetic balls produce magnetic adhesion on the ferromagnetic surfaces, which enables the soft mobile robot to climb upside-down on ferromagnetic curved surfaces. In the experiment, the inverted ceiling-based inverted crawling speed is 2.17 mm/s, and the inverted freeform surface-based inverted crawling speed is 3.40 mm/s. As indicated by the experimental results, the proposed robot has the advantages of a simple structure, low cost, reconfigurable multimodal motion ability, and so on, and has potential application in the inspection of high-value assets and operations in confined environments.

## 1. Introduction

Soft robots have emerged as a cutting-edge research field in robotics due to their inherent compliance and environmental adaptability [1,2]. By mimicking the locomotion patterns of biological organisms, such as snakes, octopuses [3], earthworms [4], and fish [5], these robots demonstrate unique advantages in complex scenarios including minimally invasive medical procedures [6], industrial pipeline inspection [7], disaster rescue operations [8], confined space operations, and deep sea environment exploration. In particular, the emerging field combining embodied intelligence and soft robotics creates opportunities for researchers to explore new innovations and applications [9].

However, existing technical solutions still face significant challenges including but not limited to material limitations, manufacturing scalability, efficient actuation, energy density and portability, adaptable shape morphing properties, and locomotive modes in real-world circumstances [10,11]. Collaboration across materials science, mechanical engineering, and artificial intelligence is critical to unlock soft robots’ potential in surgery, exploration, wearables, and beyond. Currently, most actuation methods face trade-offs. Shape memory alloy (SMA) soft robots have limited deformation ranges and usually require additional electrical systems. In contrast, shape memory polymer/composite (SMP/SMPC)-based alternatives offer either fast irreversible actuation or reversible actuation (e.g., photochemical), but with longer response times [12]. Pneumatic or hydraulic actuation systems suffer from response delays and require complex peripheral equipment [13,14]. Energy conversion efficiency in material-based actuation schemes like shape memory alloys generally remains below 35% [15]. Furthermore, limitations in single locomotion mode and inadequate morphological reconfiguration or deformation adaptability severely restrict their practical applications [16].

Magnetic soft robots based on magnetic actuation or magnetic soft materials, with its non-contact manipulation and rapid response characteristics, provide a novel approach to address these challenges [17,18]. The potential of magnetic soft robots for biomedical applications has been demonstrated in a number of impressive examples, such as magnetic helical robots for targeted therapy [19], swimming shape memory magnetic microrobots for vascular embolization [20], magnetic nanostickers for controlled bioadhesion [21], miniature ferrofluidic robot for precise drugs delivery [22], as well as the ferrofluid droplets capable of pumping biofluids [23]. Notable advancements include magnetic domain programming for complex surface navigation [24], and the forward prediction and inverse design of 4D-printed active composite plates that optimize material distribution for complex shape transformations under external magnetic fields [25]. Recent developments in neodymium–iron–boron (NdFeB) magnet–elastomer composites utilizing magneto-elastic effects [26,27] have enabled precise deformation control through structural regulation. Nevertheless, current research faces several critical issues: insufficient control precision in multimodal motion transitions [28], limited magnetic adhesion for inverted climbing [29], and elevated manufacturing costs caused by auxiliary magnetization processes [30].

To explore novel solutions, this study proposes a soft reconfigurable mobile robot with multimodal locomotion based on millimeter-scale NdFeB magnetic sphere–polymer elastomer composites [31]. Basically, the primary innovations include the following:

- Deformation-controllable magneto-elastica-reinforced elastomer actuators: A magneto-elastica-reinforced elastomer (MERE) is developed through controllable inlay methodology, coupling the discrete-scale magneto-elasticity of spherical magnetic chains with elastomer elasticity. This composite retains the mechanical softness or compliance of elastic solids while exhibiting enhanced ferromagnetic properties and magneto-elastic responsiveness. Furthermore, the deformation of the magnetic-mechanics composite-based actuator can be controlled by modulating the magnetic interactions of magnetic beads.
- Good reconfigurability: Bionic morphology design principles are established to achieve three programmable configurations: linear pattern (inchworm-like crawling), parallel pattern (steering control), and triangular pattern (omnidirectional motion). Robots based on a modular strategy exhibit better adaptability to environments or tasks, and when one module is damaged, we can replace the faulty module instead of the entire robot.
- Multimodal locomotion: Multiple fundamental locomotion modes are demonstrated, including two-anchor crawling, steering-enabled crawling, and omnidirectional movement. Notably, the embedded magnetic spheres generate magnetic adhesion on ferromagnetic surfaces, enabling inverted climbing on curved ferromagnetic substrates. Unlike inverted climbing mechanisms such as capillary adhesion [32], spiny grippers [33], voltage-controlled electroadhesion [34], and vacuum-based systems [35], the proposed method simplifies the process by eliminating the need for complex gait designs, additional adhesion system, and closed-loop feedback.

## 2. Materials and Methods

### 2.1. Basic Concept of Controllable Deformation

**Figure 1:**
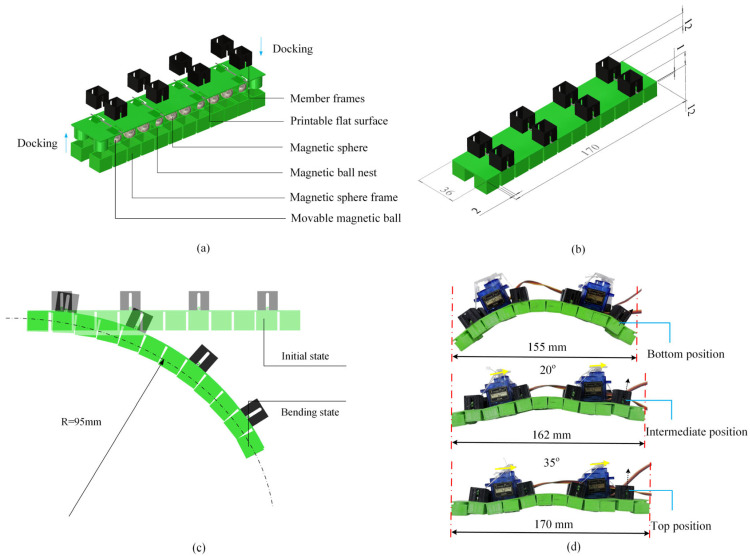
Deformation-controllable magneto-elastica-reinforced composite elastomer. (**a**) Structural assembly of a magneto-elastic soft robot cell; (**b**) the main structural dimensions of the magneto-elastic soft robot cell; (**c**) comparison of the bending state of the magnetic ball before and after assembly; (**d**) the axial elongation of a movable ball and a soft robot.

As shown in Figure 1, the basic structure of the MERE actuator is achieved by a 3D printing method. When the discrete-scale magnetic spheres are embedded into the elastic matrix, the interactions between widely spaced spheres through their magnetic fields will cause the flat elastomer to undergo bending deformation. In physics, the deformation is affected by the magnetic dipole moment, and the elasticity modulus of the elastomer. Geometrically, it is also decided by the distance between neighboring spherical magnets, length of magnetic chain, and the sectional dimension of the support structure.

It is conceivable that if the distance between neighboring spherical magnets can be modulated, the deformation of the magnetics–mechanics composites will become controllable. When all movable magnetic spheres are positioned below the neutral plane, the region below the neutral plane experiences axial compression due to magnetic attraction, while the region above experiences tension. Conversely, when all movable magnetic spheres are moved above the neutral plane, mutual attraction between spheres above the plane generates reversed bending. Therefore, we employ the rope motor-driven method to control the positions of the movable spheres and enable the deformation of the overall structure controllable. Two servo motors of MG90S (maximum torque 1.8 kg·cm) are located on the basic structure and three cables are passed through the substrates and connected to the output shaft of the motor, leading to the simultaneous deformation of the actuators during the rotation of the motor. Meanwhile, the MERE will produce a fast resilience to initial bending states once external stimuli are removed.

### 2.2. Shape Morphing Mechanism

**Figure 2:**
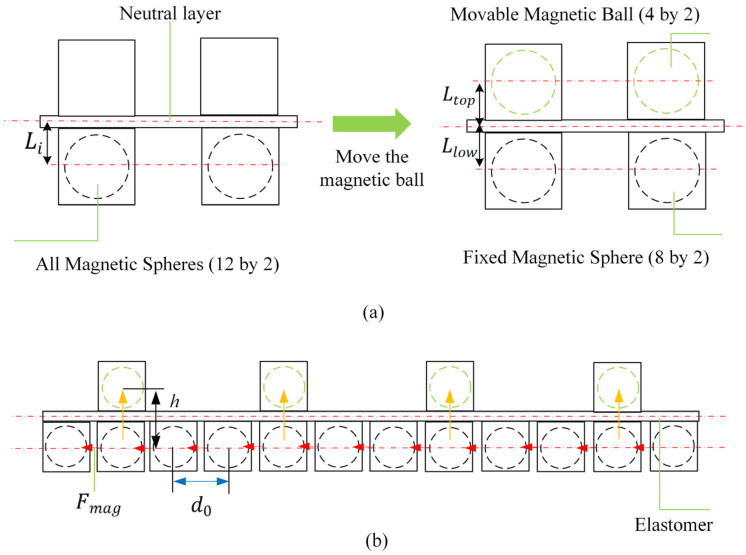
Shape morphing mechanism of MERE actuator. (**a**) Approximate cross-section of an elastomer; (**b**) magnetic elastomer and magnetic sphere movement distribution structures.

More specifically, as shown in Figure 2, after the assembly of the soft units, the pre-set internal magnetic chain structures (magnetic chains) align directionally and interact, generating mutual attractive forces. Suppose all movable magnetic spheres are positioned below the neutral plane, the region below the neutral plane experiences axial compression due to magnetic attraction, while the region above experiences tension. This induces significant axial bending moments, providing an initial deformation.

Based on the point dipole model, the magnetic moment of a single NdFeB permanent magnetic sphere is determined by

(1) $$m=\frac{BV}{\mu_{0}}$$

where the remanence *B* of the spherical magnet is 1.1 T, and the volume V of the magnetic sphere is

(2) $$V=\frac{4\pi a^{3}}{3}$$

where a is the magnetic bead radius, and the vacuum permeability $\mu_{0}=4\pi \times 10^{-7}H/M$. The magnetic moment m of a sphere with a diameter of 10 mm is approximately $0.459\;A\cdot m^{2}$. The sphere volume V is approximately $5.24\times 10^{-7}m^{3}$. The axial attractive force between two magnetic spheres is calculated using the dipole model as follows:

(3) $$F_{mag}=\frac{3\mu_{0}m^{2}}{2\pi d^{4}}$$

In the current design, the initial center-to-center distance is $d_{0}$ = 14 mm (corresponding to a surface-to-surface distance of 4 mm). The attractive force between two spheres Fmag=3.29 N. This attractive force induces a bending moment $M_{0}$ in the thermoplastic polyurethane (TPU) elastic planar body calculated as follows:

(4) $$M_{0}=n\sum_{i=1}^{11}F_{i}\cdot L_{i}\cos\theta_{i}$$

where $F_{i}$ is the attractive force between the i−th pair of magnetic beads, n is the number of columns of spheres, $L_{i}$ is the moment arm (distance from the sphere to the neutral plane of the sheet) at 5.5 mm, and $\theta_{i}$ is the angle between the magnetic force line and the deformed surface. The resulting bending moment $M_{0}\approx 0.187\;N\cdot M$. This moment drives the unit structure to overcome its own stiffness, transforming from an initial flat configuration (characterized by curvature radius *R* = ∞) to a target curved configuration (measured curvature radius *R* = 95 mm).

Furthermore, when all movable magnetic spheres are moved above the neutral plane, the mutual attraction between spheres above the plane generates asymmetric bending moments $M_{low}$ and $M_{top}$ on the upper and lower surfaces. Assuming no attraction occurs between spheres on opposite sides of the neutral plane, the resultant bending moment on the lower surface is

(5) $$M=M_{low}-M_{top}=n\sum_{i=1}^{x-1}F_{i}\cdot L_{low}-n\sum_{u=1}^{y-1}{F_{u}\cdot L}_{top}$$

where x is the number of spheres below the neutral plane and y is the number above it. The bending moment acting on the soft elastic sheet decreases to 0.116 N·M.

As shown in Figure 1c, this process clearly demonstrates the active bending capability of the soft structure based on the magnetic chain effect. The core of the vertical displacement design for the movable spheres is that it actively adjusts the spatial distribution of the magnetic field without requiring an external field, thereby changing the bending state. When a sphere moves vertically upward, the relative spatial positions between spheres change; the distance between the movable and fixed spheres increases, tending to discretize the chain-like magnetic field. This generates an asymmetric bending moment, inducing and controlling the transformation of the magneto-elastic coupling unit from a curved to a flat state.

Conversely, when the sphere moves vertically downward (i.e., when the cable is relaxed), the spheres passively recover due to the attractive force, and the soft robot returns to the curved state formed by the magnetic chains. Experiments show that when the servo motor rotates through an effective pull angle of 20°, the movable sphere is lifted to the middle of its track, and the soft unit elongates to 162 mm. At a motor rotation of 35°, the movable sphere is lifted to the top of its track, and the unit further elongates to 170 mm, achieving a maximum elongation of 15 mm.

### 2.3. Actuation Method

In constructing the actuation system, this design innovatively proposes a dual-mode actuation mechanism, as shown in Figure 1d. The deformation of the soft unit is controlled by an active shape control subsystem based on a cable–servo motor mechanism, which moves the spheres vertically upward within frame tracks. The actuation mechanism consists of a servo motor, cables, rods, and four groups of movable magnetic spheres. The cable’s elongation and friction coefficient are essentially zero. The servo motor provides a torque of 0.16 N·m at 5 V. The torsional work of the servo motor is converted into a pulling force that moves the spheres vertically upward within the tracks. Regarding the selection of the actuation mechanism, the axial attractive force between two spheres is calculated using the following dipole model (Equation (3), repeated as Equation (6) for clarity within this section):

(6) $$F_{mag}=\frac{3\mu_{0}m^{2}}{2\pi d^{4}}$$

The distance d between spheres is related to the lift height h (assuming vertical motion) as follows:

(7) $$d=\sqrt{d_{0}^{2}+h^{2}}$$

Therefore, the relationship between the magnetic force and the lift height h is

(8) $$F_{mag}(h)=\frac{3\mu_{0}m^{2}}{2\pi {\left(d_{0}^{2}+h^{2}\right)}^{2}}$$

In the actuation mechanism, the motor rotation angle $\theta_{deg}$ is converted into cable displacement via a winch (radius *r* = 0.015 m), thereby lifting the sphere according to the following:

(9) $$h=r\theta_{deg}\times \frac{\pi }{180}$$

Considering the relationship between the motor torque τ and the rotation angle or height h, under steady-state equilibrium conditions during sphere lifting, the cable tension $F_{cable}$ equals the magnetic attractive force $F_{mag}(h)\;$as follows:

(10) $$F_{cable}=F_{mag}(h)$$

The relationship between the motor torque τ and $F_{mag}(h)$ is

(11) $$\tau =F_{cable}\cdot r=F_{mag}(h)\cdot r=\frac{3\mu_{0}m^{2}r}{2\pi {\left(d_{0}^{2}+h^{2}\right)}^{2}}=\frac{3\mu_{0}m^{2}r}{2\pi {\left(d_{0}^{2}+{\left(r\theta_{deg}\times \frac{\pi }{180}\right)}^{2}\right)}^{2}}$$

Substituting the vacuum permeability $\mu_{0}=4\pi \times 10^{-7}\;H/M$, magnetic moment $m=0.459\;A\cdot m^{2}$, winch radius r=15 mm, initial center distance $d_{0}=14\;mm$, and maximum lift height for a movable sphere h=10 mm, the required motor torque for a single sphere is 0.0216 N·m. The torque required to lift four spheres is approximately 0.087 N·m. The selected servo motor, providing 0.16 N∙m torque at 5 V, satisfies the requirement for sphere movement.

### 2.4. Multiple Configurations

**Figure 3:**
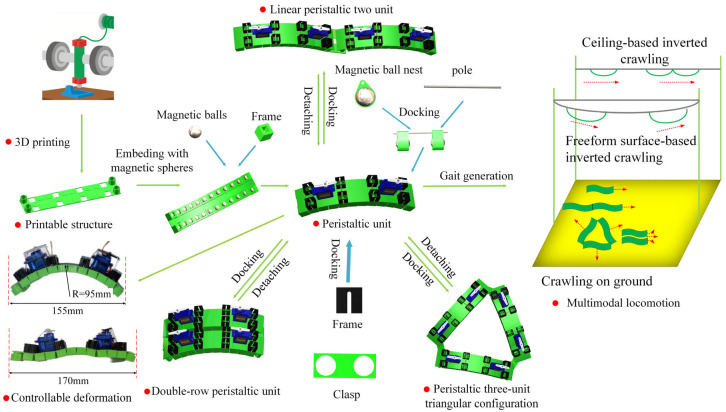
Fabrication, configurations, and multimodal locomotion of soft crawlers.

Based on an on-demand docking modular design method, multiple soft units can be flexibly assembled into morphological patterns of different dimensions. An exploratory framework is proposed, as shown in Figure 3.

In the one-dimensional morphology, the basic motion mode is the peristaltic propulsion of the unit structure. In the two-dimensional morphology, several typical configurations are derived, including a linear configuration formed by connecting units end-to-end in a single column, a parallel-shaped configuration formed by two or more columns, and a triangular topological structure formed by combining multiple units. It is noteworthy that the current morphological configurations do not cover all possible spaces, leaving potential for further optimization and expansion.

What needs to be mentioned is that spherical magnets not only generate the discrete-scale magneto-elastica to enhance the elasticity of elastomer, but also serve as distributed and stable actuation sources to drive the basic unit to recover its initial bending status. Moreover, when the robot climbs on the ferromagnetic curved surfaces, the embedded magnetic balls produce magnetic adhesion on the ferromagnetic surfaces. Therefore, whether the robot has the ability of inverted climbing is worth exploring. Excitingly, the answer given by our experiment is affirmative.

## 3. Experimental Research

### 3.1. Crawling of the Soft Crawler with Linear Pattern

**Figure 4:**
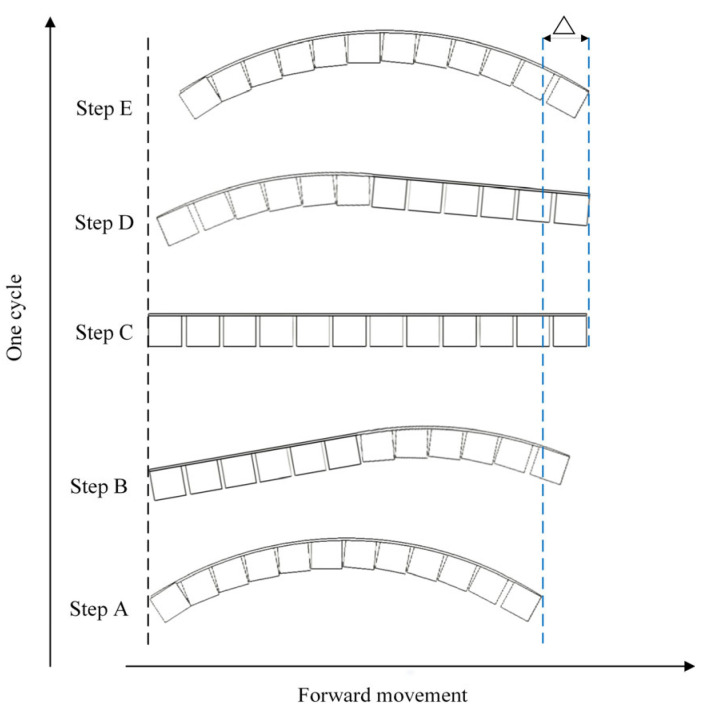
Traveling wave crawling gait of the unit structure. Step A: initial deformation state under magnetic preload; step B: asymmetric elongation through rear sphere displacement, increasing rear friction; step C: full elongation state achieving maximum stride length; step D: asymmetric contraction via passive magnetic recovery; and step E: reset to initial configuration completing one cycle.

Crawling animals, being mostly made of compliant tissues, are perfect models for mobile soft robots. Crawling occurs when the agent overcomes friction through longitudinal vibration or movement. Crawling locomotion includes several different gaits, such as two-anchor (inchworm-like) crawling [36,37], peristaltic (earthworm-like) crawling [38], and serpentine crawling [39,40]. Existing studies have shown that soft body crawling robots generally adopt a multi-segment soft structure design [41], and that the contraction–diastolic motion is achieved through the quasi-static deformation of pneumatic/dielectric elastomer actuators [42,43]. For example, a modular soft robot was proposed by Hongjun et al. [44]. The principle of two-anchor crawling has been extensively studied in caterpillars, where the animal moves forward by lengthening and shortening its body. The out-of-plane bending-based inching locomotion is usually achieved by rising and sprawling the central parts of a body that looks like an “Ω”, and the distal parts provide the anisotropic friction. The soft mobile unit proposed in this paper also adopts the motion strategy of periodic deformation, and its specific forward gait can be explained by Figure 4.

Initial deformation state (preloading phase): The system is in a stable configuration at its lowest energy state. The actuated unit exhibits a curved arcuate shape. The contact surfaces and friction at both ends are identical, providing the mechanical basis for subsequent deformation.

Asymmetric elongation phase: Under external power control, the movable spheres in the rear half of the unit migrate to the upper surface. This causes a phase change from a curved to a flat state in that region, achieving axial elongation. The contact area at the rear end increases, leading to higher friction. Simultaneously, the front half remains contracted, forming an asymmetric strain distribution that generates a net displacement driving force.

Full elongation phase: The movable spheres fully migrate to the upper surface, placing the entire actuation unit in a uniformly elongated state at maximum axial extension. Due to the higher friction at the rear end, the unit structure completes the main displacement of the propulsion phase.

Asymmetric contraction phase: The movable spheres in the rear half are controlled to migrate back to the lower surface, inducing that region to recover from a flat to a curved state. The front half remains elongated, ensuring motion continuity and reducing backward slip due to its higher friction.

Full contraction phase (reset phase): All movable spheres fully migrate to the lower surface, and the actuation unit returns to its initial curved configuration, completing one motion cycle. The cyclic execution of this process achieves directional crawling based on phase change control.

A forward gait is completed from step one to step five. The displacement per cycle ∆ should theoretically equal its axial extension value of 15 mm. However, due to incomplete recovery from the flat state to the curved state during actual deformation, the practical displacement is 9 mm.

Further theoretical analysis based on the idealized motion model for worm-like robots proposed by Kandhari et al. [45], assuming no slip, approximates the ideal velocity as follows:

(12) $$v_{ideal}=\frac{wS_{n}(S_{n}+2b)}{4n}\dot{L}$$

where w is the number of waves, $S_{n}$ is the number of moving segments per wave, b is the total number of bridging segments between a pair of moving segments within one wave, n is the total number of segments in the robot, and $\dot{L}$ is the function of the single-segment actuation rate as follows:

(13) $$\dot{L}=∆L/∆T$$

**Figure 5:**
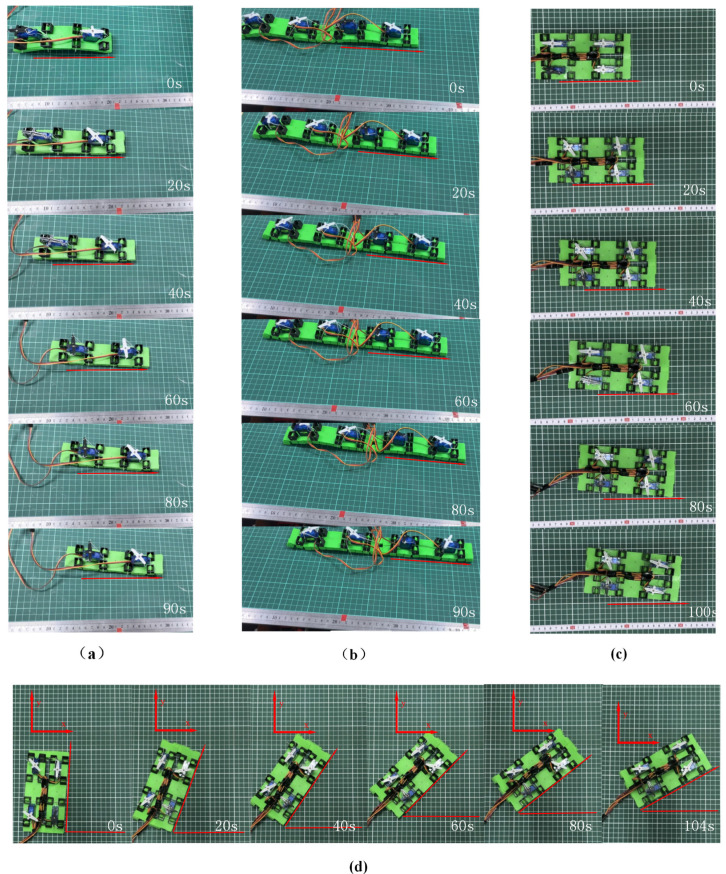
Crawling experiments of soft crawlers. (**a**) Single-unit structure crawling gait; (**b**) linear dual-unit structure; (**c**) straight-line motion; (**d**) turning motion.

According to our proposed model’s actual actuation and deformation, the waveform parameters $\left[w,S_{n},b,n,\dot{L}\right]$ for the crawling gait of a single unit are $\left[1,2,1,3,3.63\right]$. Therefore, the theoretical crawling speed for a single unit is approximately 2.42 mm/s. For comparison, the experimentally measured crawling speed (see Figure 5) is 1.11 mm/s. For a linear dual-unit configuration, the theoretical model parameters are $\left[1,2,1,5,7.26\right]$, yielding the same theoretical crawling speed of 2.14 mm/s, while the actual crawling speed is 1.05 mm/s.

Clearly, the theoretical speed overestimates the actual speed significantly. This large error arises because not all elastic energy is converted into forward work; significant energy loss occurs due to sliding friction. Therefore, we introduce an error compensation factor, the slip ratio, as follows:

(14) $$\eta_{anchor}=1-\frac{\delta_{f}+\delta_{r}}{∆L}$$

where $\delta_{f}$ is the front-end slip and $\delta_{r}$ is the rear-end slip. Experimentally, for the single-unit crawling mode, $\delta_{f}\approx$ 0.5 mm and $\delta_{r}\approx$ 2 mm. For the linear dual-unit structure, $\delta_{f}\approx$ 1 mm, $\delta_{r}\approx$ 3 mm. The predicted actual speeds are single-unit structure $v_{actual}$ = 1.66 mm/s and linear dual-unit structure $v_{actual}$ = 2.18 mm/s, where $v_{actual}$ is given by

(15) $$v_{actual}=v_{ideal}\cdot \eta_{anchor}\;$$

### 3.2. Crawling of the Soft Crawler with Parallel Two-Unit Pattern

The system adopts a parallel two-unit configuration arranged longitudinally, achieving multimodal motion through the vertical displacement control of the magneto-actuated components. In the linear motion mode, the synchronous adjustment of the vertical displacement amplitude of the magnetic driving elements in both units ensures equal-amplitude deformation characteristics during asymmetric and globally coordinated deformation phases. This establishes an isokinetic coordinated motion mechanism between the moving units ($v_{1}=v_{2}$), ultimately inducing a biomimetic crawling motion mode.

In the steering control mode, an asymmetric displacement excitation strategy is introduced, causing the magnetic components of the two units to exhibit differential displacement responses in the vertical direction. This displacement difference leads to an asymmetric strain distribution between the units ($\Delta \varepsilon =\varepsilon_{1}-\varepsilon_{2}\neq 0$). The magnitude and sign (positive/negative) of Δε determine the steering direction (left/right) and rate, satisfying the following formula:

(16) $$\frac{v_{1}}{v_{2}}=\frac{1+\beta ∆\varepsilon }{1-\beta ∆\varepsilon }$$

where it is assumed that the strain difference $∆\varepsilon =\varepsilon_{1}-\varepsilon_{2}$ is much smaller than the average strain $\bar{\varepsilon }=\frac{\varepsilon_{1}+\varepsilon_{2}}{2}$. Retaining the first-order term via Taylor expansion yields

(17) $$\beta =1/2\bar{\varepsilon }$$

**Figure 6:**
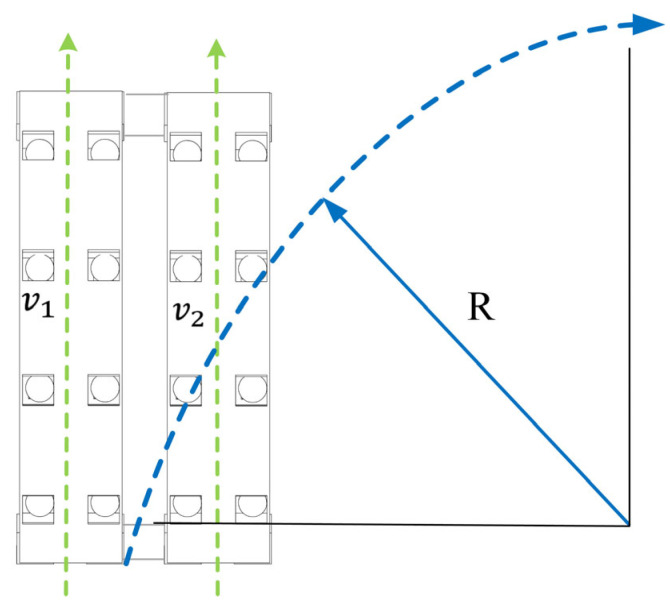
Differential crawling of parallel two-unit configuration.

This results in a differential drive effect ($v_{1}\neq v_{2}$) at the level of deformation dynamics. Experimental characterization shows that this differential drive mechanism, by adjusting the velocity gradient between the two moving units ($\nabla v=\frac{\left|v_{1}-v_{2}\right|}{p}$, where p is the unit spacing), can precisely control the system’s motion curvature radius as follows:

(18) $$R=\frac{{(v}_{1}+v_{2})\;p}{(v_{1}-v_{2})\;2}$$

thus achieving controllable heading deflection. This deformation-based differential drive method provides a motion control solution for soft robots without additional steering mechanisms, as shown in Figure 6.

The experimental results are shown in Table 1. When the parallel two-unit configuration moves at the same speed, the robot moves straight at a speed of 1.00 mm/s. When the parallel two-unit configuration moves deferentially, the soft robot turns.

### 3.3. Omnidirectional Crawling of the Soft Robot with Triangular Pattern

This study proposes a novel omnidirectional mobile soft robot system based on reconfigurable modules, with its core innovation being a closed-loop triangular topological configuration. The robot’s omnidirectional motion capability stems from the specific combinatorial control of the motion states of its three driving units. The core mechanism is that at any given time, only two deformable units execute active axial displacement (traveling wave gait), while the third unit remains stationary, acting as a bridging anchor. Through this “two-active, one-static” cooperative strategy, the robot achieves omnidirectional crawling.

#### 3.3.1. Kinematic Basis for Omnidirectional Crawling

**Figure 7:**
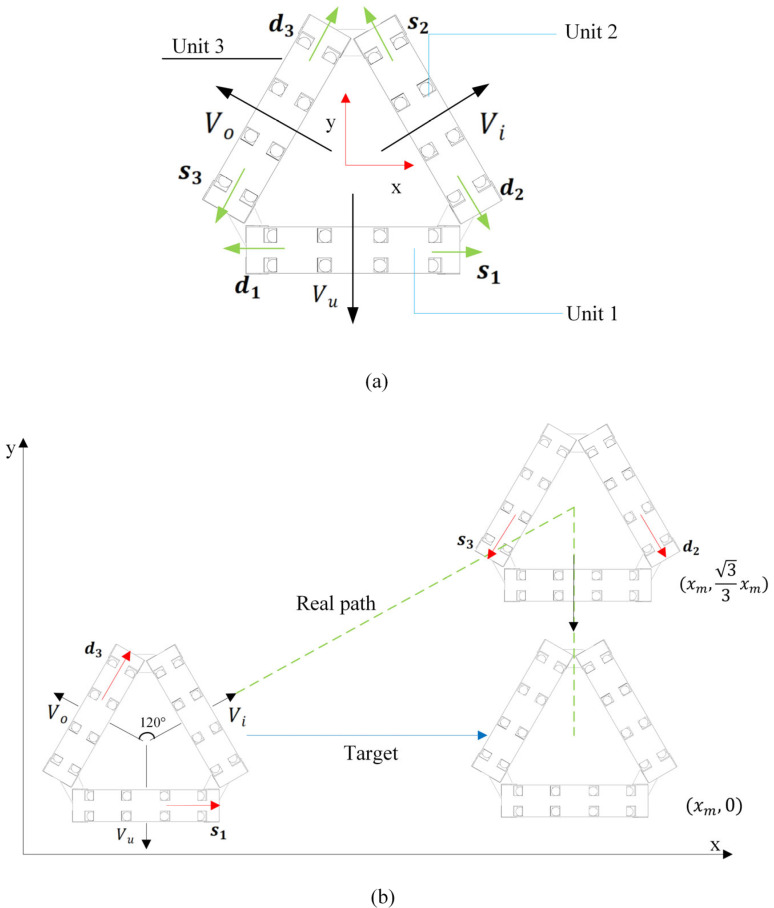
Motion of the omnidirectional mobile robot with triangular configuration. (**a**) Omnidirectional robot theoretical direction of movement; (**b**) omnidirectional robot path planning.

The robot achieves omnidirectional motion through the coordinated control of its three driving units, as shown in Figure 7. The direction vectors of each unit in a Cartesian coordinate system, with the robot’s center as the reference point, are defined as follows:

- Unit 1 movement direction: $s_{1}=(1,0)$, $d_{1}=(-1,0)$.
- Unit 2 movement direction: $s_{2}=(-\frac{1}{2},\frac{\sqrt{3}}{2})$, $d_{2}=(\frac{1}{2},-\frac{\sqrt{3}}{2})$.
- Unit 3 movement direction: $s_{3}=(-\frac{1}{2},-\frac{\sqrt{3}}{2})$, $d_{3}=(\frac{1}{2},\frac{\sqrt{3}}{2})$.

The global velocity of the robot is synthesized kinematically from the direction vectors of the individual units as follows:

(19) $$\begin{array}{c} V_{u}=(v_{ux},v_{uy})\\ V_{i}=(v_{ix},v_{iy})\;\;\\ V_{o}=(v_{ox},v_{oy}) \end{array}$$

Decompose the robot’s global velocities $V_{u},V_{i},V_{o}$ into linear combinations of the fundamental directions of two units as follows:

(20) $$\left\{\begin{array}{c} V_{u}=v_{unit}\cdot s_{3}+v_{unit}\cdot d_{2}\\ V_{i}=v_{unit}\cdot s_{1}+v_{unit}\cdot d_{3}\\ V_{o}=v_{unit}\cdot s_{2}+v_{unit}\cdot d_{1} \end{array}\right.$$

Substitute the measured crawling speed of the single-unit robot ${\;v}_{unit}=1.11\;mm/s$, and yield the omnidirectional mobile robot velocity vectors: $V_{u}=(0,-1.92)$, $V_{i}=(1.67,0.96)$, and $V_{o}=(-1.665,0.96)$. The theoretical speed of the omnidirectional mobile robot is 1.92 mm/s.

#### 3.3.2. Omnidirectional Motion

**Figure 8:**
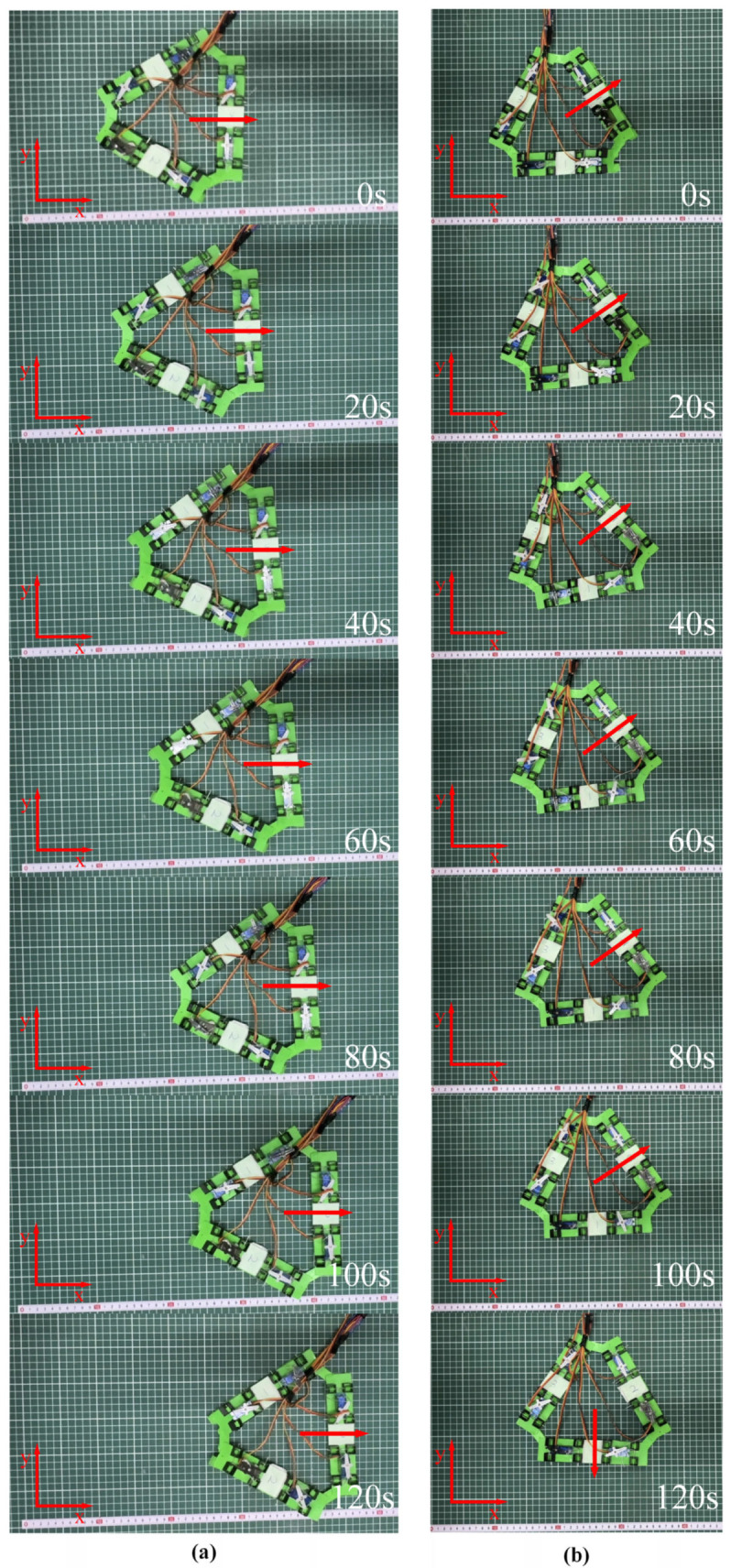
Omnidirectional crawling (**a**,**b**) of the soft robot with triangular configuration.

This configuration consists of three independently driven units spatially distributed at 120° symmetry, enabling omnidirectional motion within a plane. Each driving unit moves along its axis according to the previously described periodic traveling wave gait (including the five phases as shown in Figure 4). To achieve the goal of net translation along the global X-axis positive direction, this study designs an omnidirectional motion experiment. This motion decomposes the target displacement in the global coordinate system (X-axis translation by distance $x_{m}$) and maps it to two sequential degrees-of-freedom motions in the robot’s body coordinate system as follows:

First, drive the robot to translate at velocity $V_{i}$ for a specific distance $\frac{\sqrt{3}}{3}x_{m}$, subsequently translate at velocity $V_{u}$ for a specific distance $\frac{\sqrt{3}}{6}x_{m}$ (see Figure 8). This decomposition process effectively utilizes the directional translation characteristics induced by the “two-active, one-static” cooperative actuation, transforming complex global target displacement into a sequential combination of two orthogonal (120° angle) motions within the body coordinate system. This strategy can precisely achieve net translation along the desired X-axis.

The robot’s omnidirectional motion capability stems from the specific combinatorial control of its three driving units’ motion states. The core mechanism is that, at any given time, only two adjacent units execute active axial displacement (traveling wave gait), while the third unit remains stationary, acting as a bridging anchor. Through this “two-active, one-static” cooperative strategy, the constraint provided by the stationary unit couples the motion of the active units, synthesizing three fundamental translational directions ($V_{u}$*,*$V_{i}$*,*$V_{o}$) distributed at 120° around the robot’s center reference point, as experimentally shown in Figure 8, with specific details as follows:

- When Unit 2 and Unit 3 cooperate to displace along directions s3 and d2, Unit 1 remains stationary; the robot moves at velocity $V_{u}$.
- When Unit 1 and Unit 3 cooperate to displace along directions $s_{1}$ and $d_{3}$, Unit 2 remains stationary; the robot moves at velocity $V_{i}$.
- When Unit 1 and Unit 2 cooperate to displace along directions s2 and d1, Unit 3 remains stationary; the robot moves at velocity $V_{o}$.

By coordinating the traveling wave phases of its three actuation units, this triangular topological structure can effectively synthesize continuous motion in any direction within the plane. The motion speed along each fundamental direction ($V_{u}$, $V_{i}$, $V_{o}$) was tested at 1.25 ±0.12 mm/s, which is lower than the theoretical prediction of 1.92 mm/s. During the coordinated movement of the two units of the robot, the stationary unit, while functioning as an anchor point, simultaneously increases the overall weight of the robot. This leads to the inability of the omnidirectional mobile robot’s power to be fully converted into effective locomotive work. The core reason for this phenomenon is the neglect of “additional frictional losses from the static anchor”. By introducing the anchor slip ratio, under the triangular configuration, the front anchor slip displacement $\delta_{f}$= 0.7 mm and the rear anchor slip displacement $\delta_{r}$ = 1.1 mm. Consequently, the velocity of the omnidirectional configuration robot is calculated to be 1.42 mm/s, which is close to the measured locomotion speed. The proposed peristaltic soft robot based on the three-unit triangular configuration successfully achieves an efficient and stable planar omnidirectional crawling capability through coordinated interpolated motion in three 120° directions. This “two-active, one-static” actuation scheme not only significantly enhances motion stability but also provides a novel and highly promising paradigm for biomimetic motion design in soft robots.

To contextualize its performance characteristics, a preliminary comparison was conducted against several representative robot designs reported in the literature (see Table 2). The presented robot achieves a wave crawling locomotion at 1.25 mm/s which is comparable to the dielectric elastomer (1.32 mm/s) and magnetron–hydrogel (1.07 mm) systems. Crucially, it distinguishes itself through ultra-low fabrication costs (elastomer-based) and simplified actuation architecture. While all compared robots operate within a narrow velocity band (1.07–1.32 mm/s), the presented design demonstrates a distinctive characteristic of an ultra-low cost. While its absolute velocity falls within the mid-range of the compared systems, this performance is attained under a significantly lower cost and complexity constraints. The integration of software adaptability, basic locomotion capabilities, and low manufacturing costs suggests that this approach has the potential to develop robotic platforms for applications that prioritize cost effectiveness, ease of deployment, and environmental resilience, especially when envisaged for large-scale implementation or operation in resource-constrained environments. For such scenarios, this design paradigm deserves further study.

### 3.4. Inverted Climbing on Ceiling of the Soft Crawler

As an important branch of specialized robotics, magnetic adhesion robots achieve reliable attachment to metal substrate surfaces based on magnetic adhesion principles. They are widely used in industrial fields such as shipbuilding, bridge engineering, and building maintenance, playing key roles in scenarios like welding, surface coating, and non-destructive testing and evaluation. Currently, magnetic adhesion robot technology encompasses various configurations classified by locomotion mechanism: wheel-driven, tracked propulsion, and legged walking types [47]. Leveraging their distinct movement methods, these robots can move autonomously on planar, curved, and metal freeform surfaces, efficiently positioning themselves to designated work areas, demonstrating good environmental adaptability and task execution capabilities.

**Figure 9:**
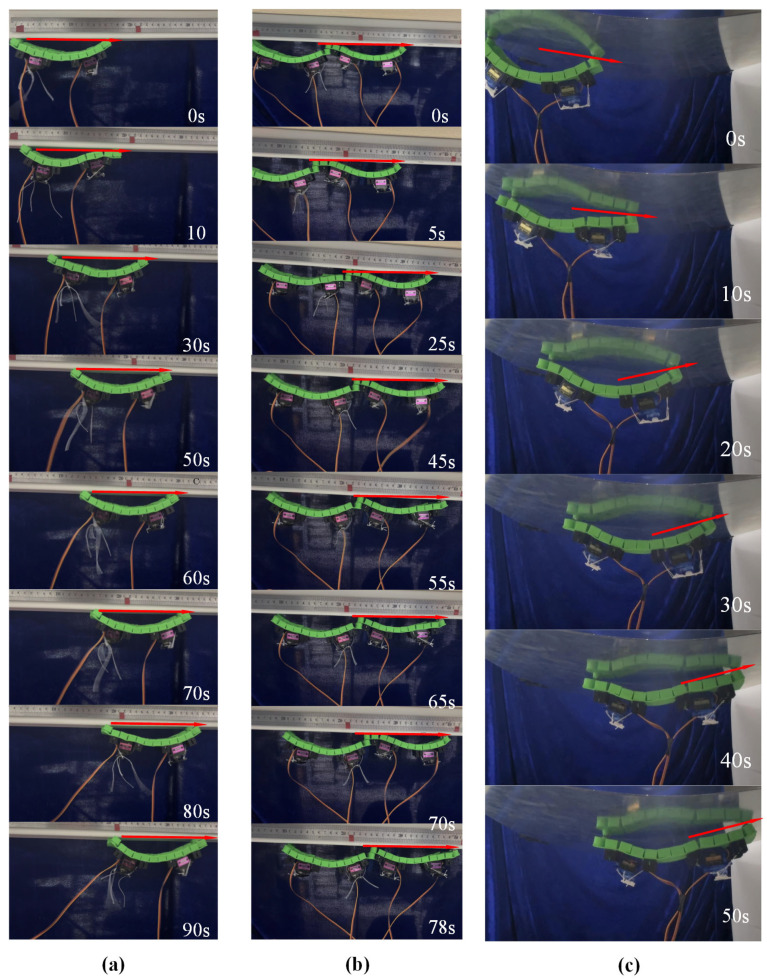
Magnetic upside-down climbing. (**a**) Unit magnetic adhesion crawling; (**b**) dual-unit magnetic adhesion crawling; (**c**) inverted magnetic adhesion climbing on a curved surface by the unit structure.

The magnetic adhesion robot proposed in this paper achieves technological breakthroughs in adhesion performance and motion modes, as shown in Figure 9. Its magnetic adhesion system is optimized for excellent climbing stability, maintaining reliable adhesion in vertical or inverted conditions. The robot employs a flexible mechanism design, achieving inverted movement on metal surfaces by precisely controlling its own bending deformation based on peristaltic crawling principles. This provides an innovative solution for tasks in special working conditions. This technological breakthrough not only expands the application boundaries of magnetic adhesion robots but also offers a new technical pathway for automated operations in complex environments.

The adsorption force of spherical magnets on ferromagnetic objects is influenced by many factors, especially the actual contact area; even if the air gap is 0.1 mm, the adsorption force can be attenuated by more than 30%. In addition, in our experiment, the curved Q235-type steel sheet with a thickness of 1.00 mm cannot make the magnetic circuit not closed, which will also lead to a significant decline in adsorption force. Therefore, we use the tension meter to test the real adsorption force of the magnetic spheres. The testing results show that the magnetic adsorption force of one unit with four magnetic spheres is 2.2 N, and that the magnetic adsorption force of the double-unit structure’s six magnetic spheres is about 3.6 N. Without changing the basic structural configuration, both the proposed single-unit structure and its in-line dual-unit configuration can achieve inverted adhesion motion. The experiments show that the system’s magnetic adhesion force effectively overcomes the robot’s own weight (single unit mass 130 g, dual-unit mass 260 g), enabling stable adhesion and directional crawling on metal surfaces while maintaining good mobility.

To quantitatively evaluate the motion performance of different configurations, a comparative analysis of the crawling speeds of the single- and linear dual-unit structures was conducted. The experimental data show that the magnetic adhesion crawling speed of the single-unit structure reaches 2.17 mm/s, while the speed of the linear dual-unit structure is 1.28 mm/s. It is noteworthy that the linear dual-unit structure uses a connection-free assembly method relying solely on spherical magnet adhesion. While this flexible connection simplifies the mechanical structure, it increases the overall magnetic adhesion force.

The speed difference primarily stems from the following mechanism: the dual-unit structure suffers from a reduced drive efficiency due to the excess adhesion force generated by the additional magnets, which manifests as increased friction resistance at the contact surface. However, the power efficiency analysis reveals that the energy conversion efficiency of the dual-unit structure (η = 75%) shows no significant decrease compared with the single-unit structure (η = 78%), indicating that it still maintains an advantage in motion efficiency.

### 3.5. Inverted Climbing on Curved Surfaces of the Soft Crawler

The surface adhesion soft robot not only achieves stable inverted magnetic adhesion climbing on flat metal surfaces but also maintains a crawling speed of 3.4 mm/s on a curved substrate with a radius of curvature of 30 cm, as shown in Figure 9. Notably, this speed is significantly higher than its baseline planar motion speed of 1.11 mm/s. Compared with traditional curved surface crawling robots relying on multi-degree-of-freedom braking systems, this design achieves a 206.31% speed increase by simplifying the actuation mechanism. This phenomenon reveals the unique motion efficiency mechanism of the flexible magnetic suction robot in the curved environment. The velocity gain of the inverted adsorption of the curved magnetic suction can be attributed to the dynamic optimization of the contact friction state, and the increase in the bending response speed caused by gravity and the redistribution of the magnetic attraction and gravity components of the robot reduces the overall static friction resistance when on the convex surface. Therefore, the robot can break through the static friction threshold at the far end and complete directional movement.

This motion strategy, based on the intrinsic deformation of flexible materials, successfully avoids the mass increase and energy loss problems associated with complex actuator designs. To the best of the authors’ knowledge, this is the first lightweight soft robot with a mass of 130 g to achieve surface-adaptive magnetic adhesion crawling, with a specific locomotion efficiency of 26.15 μm/(s·g). This innovative design provides a new paradigm shift for robots operating in curved environments, showing engineering application potential, particularly in surface inspection and maintenance within confined spaces.

### 3.6. Locomotion Capability Assessment of the Soft Crawler

In the domain of soft robotics research, the evaluation of locomotion performance across heterogeneous designs necessitates the implementation of dimensionless parameters, with dimensionless velocity constituting a critical metric. The robot’s running speed can be expressed as body lengths per second (BL/s) as follows:

(21) $$V_{body}=\frac{V}{L_{body}}\;$$

As shown in Table 3, we calculated the velocity and dimensionless velocity of the proposed soft robot in different movement modes.

In addition, as shown in Table 4, we compare the dimensionless velocity of our proposed soft robot with other reported soft robots. Our soft robot, especially in the curved ceiling crawling mode, shows remarkable performance in terms of dimensionless velocity and specific locomotion efficiency. Compared with the pneumatic soft crawling robots shown in [38] which has a dimensionless velocity range of 0.005–0.01 BL/s, our soft robot achieved 0.0219 BL/s in the curved ceiling scenario. Similarly, in contrast to the small-sized magnetically actuated soft robots investigated by [18], which had a dimensionless velocity in the range of 0.01–0.02 BL/s, our design demonstrates improved locomotion capabilities.

## 4. Conclusions

This research originates from a type of magneto-elastica-reinforced elastomer which is achieved by embedding millimeter-sized spherical NdFeB magnets into a polymer elastomer. Then, the primary innovative exploration is that the controllable deformation of the magnetic–mechanics composite-based actuator is achieved by modulating the magnetic interactions of magnetic beads. Although the rope motor-based moving magnetic spheres method appears mechanically complex, it successfully and dynamically couples the discrete-scale magneto-elastica of spherical magnet chains and the elasticity of an elastomer. A second key contribution of this work is the soft modular robot with a triangular configuration that achieves omnidirectional motion. Undoubtedly, omnidirectional moving capacity enhances the moving efficiency and maneuverability of low-speed soft robots. Crucially, this research proves an initial motivation of starting the research, that is, the proposed soft robotized configuration can climb upside-down on ferromagnetic freeform surfaces. Although the adhesion mechanism still is magnetism, it must be mentioned that it is highly challenging for a single robot to achieve multimode locomotion because it must simultaneously meet two competing and even conflicting criteria. Therefore, the proposed multimodal robots with inverted climbing on freeform surfaces and crawling on the ground capabilities will hopefully bring further advancements in robotic climbing solutions.

Clearly, some limitations of the current work also highlight opportunities for future research. Owing to the coupling complexity among the discrete-scale magneto-elastica of spherical magnet chains and the continuum elasticity of elastomer, the controllable deformation of the magnetic–mechanics composite-based actuator is limited to a simple mode. The controllable deformation method of shapes with a complex curvature still needs further study. For instance, biomimetic gradient stiffness structures can be explored to enhance deformation controllability. Furthermore, the current robot design does not consider the robot’s perceptual ability and maneuverability, which limits its application in real scenarios. Constructing multimodal perception networks (e.g., magnetic field/strain sensing) and intelligent control algorithms will significantly enhance the system’s operational capabilities in unstructured environments, providing a new technological paradigm for the field of specialized robots.

## Figures and Tables

**Table 1:** Wave characteristics, speed, and specific locomotion efficiency of the presented soft crawlers in different configurations.

Parameter	One Unit	Linear Two-Unit	Parallel Two-Unit
Total number of segments (n)	3	5	5
Actuating segments per wave ($S_{n}$)	2	2	2
Bridged segments (b)	1	1	1
Number of waves (w)	1	1	1
Front-anchored slip (mm)	0.50	1.00	0.80
Rear-anchored slip (mm)	2.00	3.00	2.00
Slip ratio (%)	68.75	75.00	82.50
Specific locomotion efficiency (mm/(s·g))	0.009	0.004	0.0038
Predicted actual velocity (mm/s)	1.66	2.18	2.39
Measured velocity (mm/s)	1.11	1.05	1.00

**Table 2:** Configuration, locomotion mode, cost, and movement speed of different omnidirectional mobile robots.

Robot	Actuation	Locomotion Mode	Materials	Cost	Velocity (mm/s)
The presented robot	Rope motor actively and magneto-elastica-driven resilience	Wave crawling	Elastomer	Low	1.25
Robot in [38]	Dielectric elastomers	Crawling	Dielectric elastomers	Medium	1.32
Robot in [46]	Magnetron	Scrolling	Hydrogel	Medium	1.07

**Table 3:** Velocities of the presented robots in different configurations and crawling modes.

Configuration	Locomotion Mode	V(mm/s)	$V_{body}$(BL/s)
Single unit	Crawling on the ground	1.11	0.0072
	Ceiling-based magnetic crawling	2.17	0.0140
	Ceiling-based crawling(curved structures)	3.40	0.0219
Linear two-unit	Crawling on the ground	1.05	0.0339
	Ceiling-based magnetic crawling	1.28	0.0041
Parallel two-unit	Crawling on the ground	1.00	0.0032
	Ground-based turning	1.11	0.0036
Triangular unit	Crawling on the ground	1.25	0.0081

**Table 4:** Locomotion capability of different soft crawlers.

Robot	Actuation	$V_{\mathrm{body}}$(BL/s)	Terrain Adaptability
The presented robot	Rope motor method and magneto-elastica-driven resilience	0.0219 (Inverted crawling) 0.0072 (Crawling on ground)	Ground Freeform ferromagnetic surface
Pneumatic soft crawling robot [38]	Pneumatic	0.005–0.01	Ground
Small-sized magnetically actuated soft robot [18]	Magnetic	0.01–0.02	Ground
Ferromagnetic soft robot [48]	Magnetic	0.006–0.015	Ferromagnetic surface
Shape memory alloy-actuated robot [14]	Thermal	0.003–0.008	Ground

## Data Availability

All data generated or analyzed during this study are included in this article.
